# Cultivating Native American scientists: an application of an Indigenous model to an undergraduate research experience

**DOI:** 10.1007/s11422-017-9850-0

**Published:** 2018-03-12

**Authors:** Tracey R. McMahon, Emily R. Griese, DenYelle Baete Kenyon

**Affiliations:** 1 Population Health, Sanford Research, Sioux Falls, SD, USA; 2 Department of Pediatrics, Sanford School of Medicine, University of South Dakota, Vermillion, SD, USA

**Keywords:** Undergraduate research, Circle of Courage, Indigenous pedagogies, Postsecondary education, Native American, Scientists

## Abstract

With growing evidence demonstrating the impact of undergraduate research experiences on educational persistence, efforts are currently being made to expand these opportunities within universities and research institutions throughout the United States. Recruiting underrepresented students into these programs has become an increasingly popular method of promoting diversity in science. Given the low matriculation into postsecondary education and completion rates among Native Americans, there is a great need for Native American undergraduate research internships. Although research has shown that Western education models tend to be less effective with Native populations, the implementation of indigenous epistemologies and pedagogies within higher education, including research experiences, is rare. This study explores the applicability of a cognitive apprenticeship merged with an indigenous approach, the Circle of Courage, to build a scientific learning environment and enhance the academic and professional development of Native students engaged in an undergraduate research experience in the health sciences. Data were drawn from focus groups with 20 students who participated in this program in 2012–2014. Questions explored the extent to which relational bonds between students and mentors were cultivated as well as the impact of this experience on the development of research skills, intellectual growth, academic and professional self-determination, and the attachment of meaning to their research experiences. Data were analyzed via deductive content analysis, allowing for an assessment of how the theoretical constructs inherent to this model (belonging, mastery, independence, and generosity) impacted students. Findings suggest that engaging Native students in research experiences that prioritize the needs of belonging, mastery, independence, and generosity can be a successful means of fostering a positive learning environment, in which students felt like significant members of a research team, developed a greater understanding and appreciation for the role of science in education and its various applications to socially relevant health issues, made more informed decisions about a career in research and the health sciences, and worked toward improving the health and well-being of others while also inspiring hope among their people back home. This study represents an extension of the application of the Circle of Courage to an undergraduate research experience and provides evidence of its ability to be used as a framework for cultivating Native scientists.

The purpose of this paper is to present an Indigenous pedagogical model and its practice within the educational space of a research experience. More specifically, the following study is an example of how an Indigenous learning model was applied to a research training program for Native American undergraduate students, with a particular focus on the impact of this model (as it was incorporated into this program) on learning outcomes and the mentor–mentee relationship. The overall goals of the program are to provide Native American undergraduate students with opportunities to interact and engage with a diverse network of interacting scientists (each working from a variety of epistemological orientations), build a supportive and inclusive learning environment that facilitates growth in students’ cognitive understandings and autonomous learning strategies, and strengthen students’ academic motivation and focus. The long-term goal is to increase the number of Native Americans who pursue careers in the health science professions and/or who conduct research pertinent to minority health and health disparities. This article may be of particular relevance to those interested in the ongoing dialogue regarding effective mentoring strategies with Indigenous students, particularly those students interested in pursuing careers in science and research.

With few published studies exploring the incorporation of Indigenous pedagogical models into research experiences for undergraduate students, this study is unique in its application of such a model to a research training program. The Circle of Courage, a positive youth development model rooted in Indigenous spiritual values and educational practices, is based on the understanding that for healthy developmental growth to occur, the needs for belonging (relational attachment and significance), mastery (competency and achievement), independence (self-determination and responsibility), and generosity (selfless giving and concern for others) must be fulfilled. Given the extensive use and successful application of the Circle of Courage combined with its relevance to Native peoples, this model provided the impetus for the development and implementation of the Summer Undergraduate Research Experience (SURE). The SURE program is a 10-week internship in which students gain hands-on research experience and mentoring in a particular field of science, in this case, the biomedical and social-behavioral health sciences.

To situate the SURE program within the broader cultural context of science education in the United States (U.S.), where this program was implemented, a brief overview of the history of Native American education and the educational system today is warranted. The re-emergence of Indigenous science is also described along with approaches to integrating Indigenous ways of knowing with Western science. The underlying principles and the development of the Circle of Courage model as well as its application to the SURE program are outlined along with the accompanying conventional qualitative methodologies used. Results describe the overarching themes of focus group narratives and a discussion of findings as they relate to Indigenous pedagogies and scientific collaborations and future directions are detailed. Because study participants were recruited from universities throughout the U.S. and many of the mentors’ primary schooling also took place at various universities throughout the country and abroad, the historical, cultural, and political context of science education is described at the national level in this paper as opposed to a more localized account.

## Locating ourselves within our research

In the pursuit of objectivity, there has been a long tradition of removing scientists, as the individuals who are investigating and applying science, from the narratives of science. In doing so, we neglect to account for the ways in which the observer plays a critical role in perceptions of what or who is being observed. Rather than being simply a dichotomy between the observer and the observed, our histories and relationships play a critical role in the collective process of knowledge production. The following is a brief summary of our stories; who we are, where we come from, and how we locate ourselves within our research.

### 

#### Tracey McMahon, MS

Both my mother and father are primarily Irish, with ethnic mixtures from other European countries, most of which can be traced to the British Isles. My Irish ancestors come from the Thomond sept of the McMahons in the province of Munster, in County Clare. McMahon is an Anglicized form of the Old Gaelic Mac Mathghamha (translated as “son of bear”).

Though we moved around a bit when I was little, most of my formative years were spent in Pierre, South Dakota, which is approximately 20 miles from tribal lands, specifically, the Lower Brule Reservation. During the summer months, we spent a great deal of time visiting extended family, particularly my dad’s family farm located four miles away from the Flandreau Santee Sioux Reservation. My experiences from having relatives living near a reservation coupled with my experiences growing up in Pierre exposed me to reservation life and racial prejudice at a young age. However, these experiences also taught me the value of family and community. From as far back as I can remember, I have always had a strong desire to effect positive social change and promote equity and justice. The racial prejudice directed toward Native Americans I witnessed growing up fueled my desire to diffuse such prejudicial attitudes and reduce disparities, a commitment that played a pivotal role in my educational and professional journey.

In working for a Native American owned engineering company while completing my undergraduate degree, I did a lot of travelling to the reservations throughout South Dakota. This experience opened my eyes to some of the difficulties encountered on reservations, the many strengths and resiliency of Native peoples, and the diversity between and within tribes. For the past seven years, I’ve been working as a research associate at Sanford Health, the largest rural, not-for-profit health care system in the United States. I am the internal evaluator of the SURE program and led the development and facilitation of the focus groups conducted as part of this study and assisted with the data analysis. As an evaluator, I often work in a supportive capacity, working closely with our community and tribal partners to understand their vision and what they hope to achieve through the services they offer or plan to offer, what they see as the role of evaluation in exploring the value and impact of their services and activities, provide evidence to inform continuous quality improvement efforts, and disseminate findings to community members and stakeholders. I’ve been fortunate to have had opportunities to continuously build relationships with the local tribes throughout my adult life, not only professionally, but also personally as I’ve come to know the students and colleagues I’ve had the pleasure of working with over the years, some of whom have become close friends.

#### Emily Griese, PhD

I grew up in rural South Dakota, a primarily Caucasian community positioned just 20 miles from reservation land, including several communities and small schools. Growing up, I was naïve to the discrimination and difficulties facing the children and families close to me, those I played alongside in athletics and thought alongside in academic bowls. As I began to understand the various factors impacting the youth within my rural community and those living so close, I was in awe of the resilience these youths showed in the face of extreme adversity. As I moved outside of South Dakota for my undergraduate and graduate education, I recognized the unique position I was in—both in being fortunate to have access to education, but even more, the experiences I had growing up to provide context and a new place for learning within my career. At this crucial point in my life and education, I was able to move back to South Dakota to work with an organization and team that truly embraced and engaged in community-based participatory work across the region, both in rural and tribal communities. In working with Dr. Kenyon and her team as a postdoctoral fellow, I was able to establish and grow relationships with community members and learn alongside as we worked to tell the stories of these communities. From this work came the present study in which I led the qualitative data analysis. My research continues in this area, working to uncover and better understand the unique sources of resilience among Native American and rural youth.

#### DenYelle Baete Kenyon

I grew up in the country surrounded by my uncles’ farmland outside of Sioux Falls, South Dakota. It was only after I moved away and went to college that I fully appreciated the advantages I was afforded growing up in a stable loving home, and realized that many others were not so fortunate. After I discovered my love for research as an undergraduate and decided to go to graduate school (which was unheard of in my extended family), my mission became to make an impact beyond the ivory tower through research. During my postdoctoral fellowship, I heard there was a major research emphasis in my hometown area and was ecstatic it was in health disparities research with Native American populations. It was only after I left South Dakota, living near tribal nations in Minnesota and Arizona, that I realized the atrocious disparities faced by Native American people in my own home state. I was excited yet intimidated to tackle these topics firsthand, as there were only a handful of Native American students who attended my school growing up.

I knew I wanted to work with teens, and having learned about the importance of community-based participatory research principles, my first projects were conducting Photovoice projects with Native American teens from a tribal school and Native American freshman college students. I have always believed education was integral to empowerment, and in reflecting on the importance of the path my undergraduate research internship started me down, I started a research internship for Native students through the Pathways into Public Health program in 2009. I feel deeply about promoting social justice and making an impact with Native American students, giving them the inspiration and research experience I received in college. I have felt firsthand the value of having Native students on our research projects and learning from them (not just them learning from us). For example, the Native students involved in these projects have taught me a great deal about traditional Native cultures and some of the identity issues that come with walking in multiple worlds.

In approaching the conduct of research with people from a culture different than my own, I received great advice from our cultural liaisons to know and be true to myself, not to posture or pretend to know more about the culture than I actually do. After doing this work for almost 10 years, I can’t imagine not working toward Native American causes both in my personal and professional life. This underlying value of diversity, respect, and social justice for our First Americans is what I try to instill into my two young children. When we were starting to build SURE, the first author, Tracey McMahon, and I were in discussions with Sarah Brokenleg, a partner working on related efforts for Native undergraduates in healthcare careers. She suggested using the Circle of Courage model, which I previously read about but had not thought about applying to an older group of students. Her father, Dr. Martin Brokenleg, is one of the co-creators of the Circle of Courage. To become more familiar with the model, we received training on the Circle of Courage and attended a number of presentations by Martin Brokenleg and his colleagues in which they spoke about the development and underlying principles of the model. We have been applying the model for over seven years and have presented on our use of the Circle of Courage in several local and national contexts. We are grateful for this opportunity to share some of our experiences throughout this journey and hope the reader enjoys this small part of our story and can apply some lessons learned to their own work.

## A brief history of education with Native peoples in the United States

To better understand Native student persistence in higher education, it is important to recognize the conflicted relationship Native peoples have had with education in the U.S. post-colonization. In the early 1800s, the U.S. government instituted policies aimed at acculturating and assimilating Native Americans, which resulted in a systematic assault on Native cultural identities. These policies used education as an instrument for acculturating Native youth to Eurocentric ways of thinking and living (an ideology emphasizing European or Anglo-American historical and cultural perspectives often so as to exclude other political, geographical, or cultural groups) and reinforcing power relationships (Gundlach Graham 2012). This political era became known as the boarding school era, in which government run boarding schools carried out the “civilization” process by forcibly removing children from their homes for extended periods of time, stripping them of their traditional Native identity, and exposing them to religious indoctrination, academic and vocational training, and routinized labor (Oakes 2013). Numerous cases of sexual, physical, and emotional abuse were also reported (Margolis 2004). The culmination of boarding school experiences often resulted in feelings of rejection and alienation from both White society and Native society (Churchill 2004).

## The contemporary political and cultural context of Native American science education

Although the assimilation policies that characterized the boarding school era are no longer in place, subtle assimilation practices can still be seen within the educational system and methods of scientific inquiry today. Unlike in the past when federal education policies were explicitly aimed at assimilating and acculturating Native Americans, assimilation today may not be the goal, but rather the “inevitable outcome of education that occurs through the formal structures of Western schooling” (McKinley and Brayboy 2005, p. 437). For instance, some researchers have argued that the national movement towards the standardization of educational practices assimilates and acculturates students to the dominant culture (e.g., Forbes 2000). This movement has gained momentum in recent years since standards-based accountability has been one of the leading principals of educational reform in the U.S. over the past few decades in an effort to increase academic rigor and hold schools and educators accountable for educational progress, by which students’ scores on standardised tests are the unifying measure of student achievement (Kim 2010). Because standardized test performance are often required by schools and states for receipt of federal aid, students and teachers are pressured to abide by federal education mandates and assimilate to the academic testing culture. This uniformity in education not only discourages diversification of curricular content and methods of instruction, but also “creates problems of deculturalization and disempowerment of minority cultures and viewpoints, forcing them to assimilate into the knowledge and values of the dominant culture” (Kim 2010, p. 14). Though many educators and school administrators do not equate high test scores with quality schooling and support diversity in school curricula, pressures to align education content and standards with international benchmarks and compete in the global economy have furthered the national political agenda for a test-driven school system (Spring 2011).

It should be noted, however, that despite the use of standardized testing as a collective measure of academic competencies, education in the U.S., in many respects, is localized. The localization of education in the U.S. is largely the result of public education being a state’s responsibility. Though the federal government does provide some funding for education, 92 percent of this funding comes from state, local, and private sources (U.S. Department of Education 2017). Rather, the federal government effects education through legislation. The federal government also oversees the Bureau of Indian Education (BIE), whose mission is to provide quality education from early childhood through life “in accordance with a tribe’s needs for cultural and economic well-being” (U.S. Department of the Interior 2017b). Whereas the BIE has made efforts to strengthen Indigenous cultures through pedagogy and curricula tailored to Indigenous learning needs and ways of knowing, only 7 percent of Native students attend schools administered by the BIE, while 90 percent attend public schools. This puts much of the responsibility for recognizing the unique education and cultural needs of Native students on public schools.

Therefore, state education policies tend to have a greater impact on Native students compared to federal policies (Meza 2015). These policies largely center on school and class size, school choice, student tracking, teacher education/certification and selection, teacher pay, instructional methods, curricular content, graduation requirements, infrastructure investment, and values. The lack of publicized research on the role of state education agencies as actors in science education policy (Cheek and Quiriconi 2011), coupled with the diversity in state science education standards (Lerner, Goodenough, Lynch, Schwartz, and Schwartz 2012)—which can vary between local school districts and even schools within the same district—makes it difficult to characterize the influence of local culture and politics on science education. Conversely, the influences of the dominant cultural values and norms on science and science education in the U.S. is well established in the literature and too numerous to explain in great detail. For our purposes, a brief overview of the cultural terrain Native students navigate will help frame the broader discussion of ongoing efforts to forward an epistemological pluralism inclusive of Indigenous ways of knowing.

A common phrase among Native students describing their experiences of navigating the dominant culture while simultaneously retaining their own culture and identity is “walking in two worlds” (Peacock and Cleary 1998). This ability to find balance while moving in and out of both Indigenous and non-Indigenous learning systems signifies a common theme within the educational persistence literature. Though a number of studies have shown that such conflicting cultural values as individualism versus collectivism, competition versus cooperation, and assertion versus quiet observation/listening can have potentially damaging effects on Native students’ advancement in public schools and state colleges and universities (e.g., Crawford, Cheadle, and Whitbeck 2010), an emerging body of literature is confirming what many Native peoples have known for centuries. That is, a strong cultural identity safeguards against cultural assimilation and alienation.

## The re-emergence of Indigenous ways of knowing and Indigenous science

Because how we come to know and understand the natural world is a culturally contexted social phenomenon, influenced by ideologies and to some degree determined by them, it is important to understand the role of ideology in scientific practices. Given this study’s focus on pedagogy (defined here as the process of guiding and inspiring learning or coming to know) as a framework for cultivating the growth and development of Native scientists, a brief overview of the relationship between science and ideology and comparison between Indigenous and Western knowledge systems is justified. Indigenous ways of knowing and ways of being continue to be undervalued and underrepresented in science curriculum and scientific research in the U.S. Thus, it is our hope that Indigenous and Western knowledge systems work effectively together, recognizing the legitimacy of both and seeing the strengths in each.

In the context of scientific research, epistemological expansion and ideological reform requires a critical examination of how science is defined in order to truly expand and improve scientific practices and methods of scientific inquiry. The term “ideology” was originally coined by Antoine Destutt de Tracy as the science of “intellectual faculties, their principal phenomena, and the more remarkable circumstances of their activities” (cited in Richards 1993, p. 103), or put differently, the science of our acquisition of ideas about the nature of reality. Scientific knowledge, however, is inevitably situated, historically contingent, and culturally relative. What is considered science and “scientific facts” is dependent upon who is doing the defining and the observer’s cultural worldviews, which are inevitably shaped by history, sense of place or locality, relationships, and experiences. As such, many Indigenous, feminist, and postcolonial scholars have argued that the practice of science cannot be separated from its “cultural, historical, and ideological contexts” (O’Sullivan 2012, p. 402). In other words, scientific knowledge cannot be disentangled from its roots of knowledge acquisition, not strictly from a methodological standpoint (the procedures used in a particular discipline or topic of study), but also epistemologically (the nature and origins of knowledge) and ontologically (the nature of being).

Indigenous knowledge is often specific to a tribal community or locality. It is relational to the experiences of a particular people and their long-term association with a place or what Ella Deloria (1998) refers to as a “spiritual culture area.” Therefore, it is difficult (or even impossible) to define or translate. Still, the gaining recognition of Indigenous science as being distinct from non-Indigenous science as well as the role of Indigenous science in the self-determination of Indigenous education and efforts to re-claim science and knowledge production makes it necessary to clarify its meaning. Cajete (2000) states that Indigenous science refers to “the entire edifice of Indigenous knowledge” (p. 3) and is encompassed in all the ways in which we “come-to-know” our cosmology, the natural world, and life-sustaining relationships (p. 80). Coming-to-knowing can be understood as “entering a relationship with the spirit of knowledge, with plants and animals, with beings that animate dreams and visions, and with the spirit of the people” (Peat 2002, p. 65). How we come-to-know is contingent upon a people’s cultural worldviews or paradigms. Though Indigenous paradigms can vary from one tribe or individual to another, as articulated by Little Bear (in Cajete 2000, p. x), such paradigms often embody notions of “constant motion and flux, existence consisting of energy waves, interrelationships, all things being animate, space/place, renewal, and all things being imbued with spirit,” in which the terms spirit and energy are oftentimes interchangeable. Foundational to paradigms of Indigenous science is the relational maintenance of balance, harmony, and mutual reciprocity based off of traditions of holistic participation. The ongoing epistemological and ontological processes of coming-to-know and coming-to-be are deeply relational and holistically interwoven into the journey of life (Higgins 2014).

As opposed to a focus on the relationships and interdependence of processes and energies or spirit, by contrast, Western science (also referred to as Eurocentric science) compartmentalizes knowledge, seeking to understand phenomenon through reductionism, meaning dissecting and classifying complex phenomenon in order to explain it through the examination of its individual parts. When the story of Western science meets that of Native origins, “a clash of paradigms occurs” (Cajete 2000). This is not to say that these knowledge systems are incompatible, but on the contrary, may inform and compliment these different ways of coming to know and coming to be.

## Building bridges between Indigenous and Western scientific epistemologies

‘Go into the forest, you see the birch, maple, pine. Look underground and all those trees are holding hands. We as people have to do the same.’ – Charles Labrador, Mi’kmaq Spiritual Leader, Healer, and Chief of Acadia First Nation, Nova Scotia (from an interview with Todd Labrador, quoting his father’s wisdom; Kierans 2003, May 18).

The Indigenous pedagogical model used in this study, the Circle of Courage, was developed via a transdisciplinary engagement of Western and Indigenous knowledge systems, fusing traditional child development knowledge of Indigenous peoples with Western social and behavioral sciences. As such, it is important to understand how such cross-cultural and co-learning strategies can advance scientific understandings and approaches to life-world issues. Reflective of Cajete’s (2000) call for “a mutually beneficial bridge and dialog between Indigenous and Western scientists and communities,” is the recent increase in discourse and use of Indigenous paradigms and methodologies as well as collaborations between Indigenous and non-Indigenous scholars/researchers both nationally and globally (p. 7).

The integration of Western science and traditional Indigenous knowledge (also referred to as traditional ecological knowledge) has been an increasingly popular method for involving Native American peoples and gaining community support for research projects. Though the dialogue between traditional knowledge and Western science can provide mutually beneficial scientific insights and lead to more self-reflexive practices, the process through which different knowledge systems are translated or transacted is often complicated and influenced by political dimensions and power relations. For example, as elucidated by Ahenakew (2016), the translation of Indigenous forms of knowledge and being into Western epistemological terminology and categories fails to capture the lived experiences and logic of what is being translated. Additionally, traditional knowledge can be difficult to access and socio-political conflicts can hinder integration. Such conflicts might include: how different actors use the traditional knowledge term, the agendas that are advanced or concealed through their use of the term, how beliefs and actions are constrained and directed by these meanings, and who benefits (Nadasdy 1999). While some Native communities and scholars may doubt the sincerity (if not willful obfuscation) of non-Native scientists’ desire to integrate traditional knowledge, non-Native scientists may also have doubts about the validity and efficacy of traditional knowledge or even its existence or relevance due to the drastic changes in lifestyle of Native peoples in modern times (Nadasdy 1999). Many Native peoples are also cautious of attempts to disseminate traditional knowledge, suspicious of the assumptions and intentions of Western science and appropriation of cultural concepts and ideas (Cajete 2000).

An approach to braiding together traditional knowledge with Western science in research and science curricula gaining momentum both nationally and globally is transdisciplinary research. This approach can be viewed as an opportunity for what Bartlett, Marshall, and Marshall (2012) refer to as “Two-Eyed Seeing,” or:

… learning to see from one eye with the *strengths* of Indigenous knowledges and ways of knowing, and from the other eye with the *strengths* of Western knowledges and ways of knowing, and to using both these eyes together, for the benefit of all (p. 335).

Piaget (1972), who is credited for the term’s origins, conceived of transdisciplinarity as a form of scholarship that “would not only cover interactions or reciprocities between specialised research projects, but would place these relationships within a total system without any firm boundaries between disciplines” (p. 138). In other words, transdisciplinary scholarship represents the possibility of knowledge to transcend the disciplines. Though there is no consensus on the definition of the term, it can generally be understood as an approach to the research process that integrates multiple ways of knowing from perspectives that transcend traditional disciplinary boundaries in an effort to develop innovative solutions to socially relevant issues. This approach can be especially useful when addressing research topics calling for epistemological pluralism (plurality in ways of knowing) and new or multiple analytic perspectives. As such, the approach has become progressively popular as a way of bridging Indigenous and Western sciences or “facilitating the ‘talking and walking together’ of Indigenous and mainstream sciences” (Bartlett, Marshall, and Marshall 2008). Some of the barriers and constraints to transdisciplinary engagement between Western and Indigenous knowledge systems, often involving Indigenous and non-Indigenous researchers and stakeholders, are similar to the challenges described above in relation to the integration of Western science and traditional knowledge.

As suggested by Cram and Phillips (2012), an “interstitial space” may help overcome these challenges, “whereby no one’s rules, vocabulary, ontology, epistemology and/or culture dominate” (p. 45). However, such efforts to create and work within interstitial space would need to be evaluated to determine if neutrality has been achieved and the extent to which it leads to scientific integration and innovative solutions to the issues or problems identified. Through the evaluation of the SURE program, this study investigates how various aspects of the scientific learning environment, mainly those nurtured through the Circle of Courage, can advance Native students’ cognitive knowledge and collaborate effectively with health research scientists to find new ways to prevent and treat disease and other aspects effecting human health. The assessment of an interstitial space was not a goal of the evaluation of this program, but is nonetheless important to keep in mind when interpreting results described later in this paper. Furthermore, to reduce power and privilege differentials within the SURE program, students evaluate their own needs and develop projects based on this evaluation. Students’ perspectives are also taken into account in program design and implementation through the evaluation of the program each year. Historical hierarchies that facilitate oppression are challenged with the Indigenous framework that the SURE program utilizes, which is a culmination of traditional knowledge and transdisciplinary research. Our use of an Indigenous model was not done as a means of gaining community support or a grant requirement, but rather the logical result of a collaborative partnership with a colleague and friend engaged in similar work.

## The Circle of Courage

As previously mentioned, the Circle of Courage provided the impetus for the development and implementation of the SURE program. Since the SURE program draws applicants from various tribes across the U.S., a model incorporating a variety of Indigenous worldviews was preferred over a localized or culturally-specific model. The Circle of Courage, created by Larry Brendtro, Martin Brokenleg, and Steve Van Bockern (1990/2002), represents a synthesis of Indigenous and Western knowledge and is a positive youth development approach originally developed as a philosophical framework for addressing the needs of children and youth with broken circles (those who have experienced trauma, dis-couragement, and at-risk) through the creation of reclaiming environments and cultures of respect. The Circle of Courage began as a collaboration between three professors who grew up in South Dakota and were colleagues at the time at a liberal arts institution, Augustana University, in Sioux Falls, South Dakota. Martin Brokenleg is a member of the Rosebud Sioux Tribe, more properly known as Sicangu Lakota Oyate or the “Burnt Thigh Nation,” and Larry Brendtro is an adopted member of this tribe (Rosebud Sioux Tribe 2016).

**Fig. 1:**
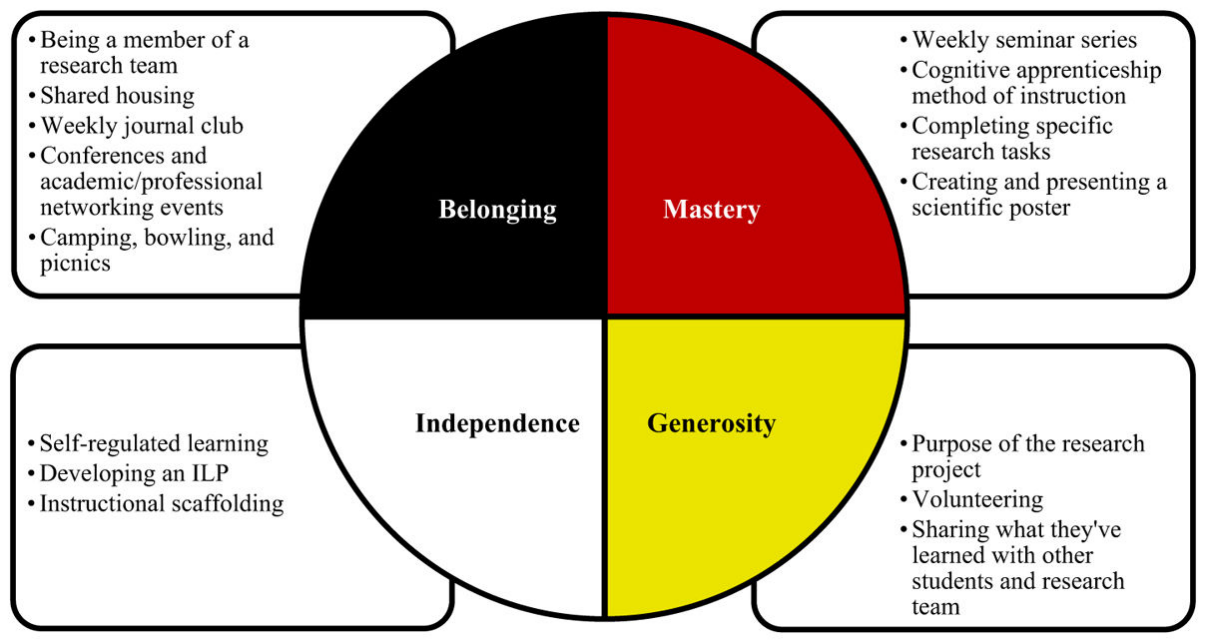
Circle of Courage needs and SURE program activities

The Circle of Courage is rooted in Indigenous spiritual values emphasizing the Lakota people’s and other Indigenous tribes’ reverence toward children as spiritual creations and “sacred beings.” The model is also guided by Indigenous spiritual ecology principles, interrelatedness/interdependence, and the spiritual dimensions that are the four foundations of the model, referring to these foundations as: the *spirit* of belonging, the *spirit* of mastery, the *spirit* of independence, and the *spirit* of generosity (Brokenleg and Van Bockern 2003). The selection of four underlying principles was intentional, as “the number four has sacred meaning to Native people who see the person as standing in a circle surrounded by the four directions” (Brendtro et al. 1990/2002, p. 45). The philosophies of the Circle of Courage are portrayed as a medicine wheel (see Fig. 1), and this symbol is foundational to its epistemology (Brendtro et al. 1990/2002, pp. 54–55). Cajete (2000) refers to the medicine wheel of the tribes from the Great Plains region as an example of the Indigenous conception of the universe, where the universe is “a circle of learning, life, and relationship that is all inclusive of all-important information needed to make life decisions” (p. 76). In reference to the medicine wheel, George Blue Bird, the Lakota artist who created the Circle of Courage art used to depict the model, explains,

… Tribal people use the circle to show that all of life must be in balance and that we all must be connected to one another. The four colors—black, white, red, and yellow—stand for the four directions, and also for the four races. We should all live in harmony, part of the same circle (Brendtro et al. 1990/2002, pp. 132–133).

The model is also integrated with evidence from modern research and the fieldwork of some of the founders of positive psychology and human development research (Brendtro et al. 1990/2002). For example, the Circle of Courage principles mirror similar concepts in Abraham Maslow’s (1971) hierarchy of human needs and Erik Erikson’s (1950) stages of psychosocial development (Brendtro, Brokenleg, and Van Bockern 2014). Notably, these seminal works, which laid the groundwork for contemporary theories of human development, were greatly influenced by anthropological studies of Native tribes, such as Erikson’s studies of the Oglala Sioux (also referred to as the Oglala Lakota) and Yurok tribes (1950) and Maslow’s studies of the Blackfoot Nation in Canada (1971). In fact, Blackfoot beliefs informed the development of Maslow’s hierarchy of needs model in such a fundamental way that it can be considered a form of cultural perpetuity, albeit some essential Blackfoot teachings relating to the model were excluded (Blackstock 2011). Given that this model is grounded in a consilience of evidence from Native American child rearing practices and Western scientific studies of human development, resilience, and neuroscience (Brendtro, Brokenleg, and Van Bockern 2013), the Circle of Courage represents a culmination of Indigenous and Western knowledge and is an approach to nurturing children and youth which transcends disciplines. For more information on the history of the development of the Circle of Courage model, see *Connecting with kids in conflict: A life space legacy* (Morse 2008).

The founders of the Circle of Courage have developed toolkits and trainings to help schools and educators cultivate belonging, mastery, independence, and generosity with their students, with indicators and descriptors demonstrating when these needs have been met (Van Bockern and McDonald 2012). Research has shown that the essentials of the model have shown measurable success with students. For example, in a case study of a magnet middle school which sought to evaluate the impact of the school’s philosophical foundation of “Engage, Achieve, and Belong” on student achievement, student belonging, and parental engagement, José Villarreal (2011) used the Circle of Courage as the lens through which the school’s core values were examined and defined for measurement. Findings indicate that this philosophical framework was a potent tool for closing achievement gaps, particularly among underrepresented groups. The model has also been effectually applied as a means of reducing risk, developing resilience, and building assets in a variety of other settings, including counseling (Frankowski and Duncan 2013), families (Garfat and Van Bockern 2010), organizations providing services to Aboriginal populations (Ambtman, Hudson, Hartry, and Mackay-Chiddenton 2010), behavior management systems (Harper 2005), treatment and juvenile justice (DeSalvatore, Millspaugh, and Long 2009), and faith-based programs (Larson and Brendtro 2000).

The Circle of Courage has been used with various Indigenous peoples throughout the world as well, including the U.S. (James and Renville 2012), Canada (Marchand 2011), South Africa (Roberts 2000), New Zealand (Espiner and Guild 2010), and Australia (Rauland and Adams 2015). In publications noting the model’s use with Indigenous populations, adaptations made in these contexts to account for different articulations of Indigeneity were not discussed. Nevertheless, the model has demonstrated success in its ability to respond to the learning needs of students and educators of diverse cultures, ages, and settings. Therefore, the model holds promise in its ability to be used as a framework for promoting academic resilience among Native undergraduate students. To our knowledge, the Circle of Courage has not been used with postsecondary students. As such, the present study extends the application of this framework to a research experience for Native American undergraduate students.

The Circle of Courage is integrated into the SURE program through various activities and components intended to promote belonging, mastery, independence, and generosity, which will be described in further detail in the following description of each of these four needs. (See Fig. 1 for examples of program activities that correspond to each of the four Circle of Courage needs.)

### Belonging

People have an innate need to belong, which motivates the desire to maintain lasting, positive relationships and has strong effects on emotional and cognitive functioning (Baumeister and Leary 1995). Consequently, it is of no surprise that, of the four developmental needs encompassed within this model, belonging is viewed as being critical to fulfilling the needs for mastery, independence, and generosity (Brendtro, Mitchell, and Jackson 2014, p. 12). Knowing that we are significant, that others care about us, is one of the pillars to belonging (Brokenleg 2012). Cajete (1994) asserts that not only are relationships the foundation to learning environments, but they are also the basis of tribal communities. Kinship often provides an expansive network of social support and belonging among Indigenous peoples which extends beyond blood relations and is exemplified in the term, mitakuye oyasiŋ, a Lakota phrase meaning *all my relations* or *we are all related*. This term is reflective of an Indigenous worldview of our interrelatedness to all of creation (Brendtro et al. 1990/2002). Interdependence and mutual responsibility are also elemental to Native ways of knowing and what Cajete (1994) describes as the essence of tribal education. Within the college setting, studies have shown social connection and belonging to be significant predictors of positive postsecondary education outcomes among Native students (Flynn, Duncan, and Jorgensen 2012).

Within the SURE program, belonging is encouraged through regular interactions between students and their mentors and research team, shared housing with program peers, journal club, group excursions to conferences and other formal academic/career advancement and networking events, and informal social gatherings such as camping, bowling, and picnics.

### Mastery

Mastery can be understood as the need to feel competent and is fostered through opportunities to learn, explore, and develop abilities and talents (Brendtro, Brokenleg, et al. 2014). It is argued that when the need to feel competent is satisfied, motivation for further achievement increases, thereby leading to further problems solving strategies and skill development (Brendtro et al. 1990/2002). Since learning often takes place within a social environment, relationships play a critical role in developing competence. For example, evidence suggests that discovering and achieving one’s potential is best achieved with the support of mentors and more skillful peers (Rieske and Benjamin 2015). Traditionally, apprenticeships played a significant role in teaching and learning within Indigenous cultures as well as experiential learning, storytelling, art, unconscious imagery, and ceremony (Cajete 1994). Learning strategies inherent to the apprenticeship model (e.g., simultaneous processing and watch, learn, then do) have also been found to be to be some of the cognitive styles and learning preferences held in common by gifted Diné, Lakota, and Ojibwe students (Gentry, Fugate, Wu, and Castellano 2014).

In the SURE program, mastery is facilitated through weekly seminars focused on academic and professional development (e.g., applying for scholarships, CV and resume building, responsible conduct in research) and health disparities research (e.g., history of research with Native peoples). Using a cognitive apprenticeship model of instruction, mastery is also promoted through the ongoing refinement of students’ research skills by means of their completion of a research project, a more in-depth understanding of the research process, and the creation of a scientific poster, which is presented to their peers, mentors, and other attending professionals and academics.

### Independence

Independence is interpreted as having agency over one’s life course and the self-efficacy to make responsible decisions (Brendtro, Mitchell, et al. 2014). Within traditional Native cultures, independence contributed to determination in that “making one’s decisions fostered motivation to attain a given goal and responsibility for failure or success” (Brendtro et al. 1990/2002, pp. 52–53). Goals were self-imposed, and the achievement of those goals was itself the reward (Brendtro et al. 1990/2002). In many Indigenous cultures, independence is balanced by social controls in which empathy and respect for others stimulates pro-social goals and behaviors (Brendtro et al. 1990/2002). In this way, autonomy is harmonized with belonging and anchored in secure attachments. Brendtro et al. (1990/2002) also explains that independence tends to evolve slowly throughout the life course, beginning with dependence and continuous guidance through instruction, modeling, and consistent encouragement and recognition of accomplishments and matures through the gradual expansion of responsibilities and the eventual discovery of one’s potential. Self-regulated learning, or the use of self-directed processes to build academic strengths, applies this idea of promoting student autonomy and agency to stimulate the growth of cognitive understandings, a method that has had measurable success with Native students. For example, Patterson, Wolf, Ahuna, Tinnesz, and Vanzile-Tamsen (2014) observed higher retention and graduation rates among Native American postsecondary students who completed a course in self-regulated learning compared to non-completers.

Self-regulated learning is built into the SURE program to nurture independence. Specifically, students meet with the principal investigator and their primary mentor at the beginning of the summer to develop an Individualized Learning Plan (ILP). ILPs are a strategy used by students—with support from their mentors and program staff—to define their research experience goals in order to plan opportunities for the student to progress toward these goals. ILPs are also used to document the knowledge and skills gained throughout the summer along with other accomplishments. Students’ progression toward meeting these self-defined goals is then assessed by the students at midpoint with their mentors and program staff to ensure they have ample opportunities to successfully accomplish these aims by the end of their research experience. Independence is also supported through instructional scaffolding in which mentors and more skillful peers help students develop research skills by providing guidance and support. Then instruction and oversight are gradually tapered off as students gain more experience and autonomous learning strategies.

### Generosity

Like mastery and independence, generosity also has communal traits. The selfless giving of time, love, possessions, and services often necessitates an empathetic affinity toward others, prosocial values, and altruistic behaviors (Brendtro, Brokenleg, et al. 2014). Moreover, the formation of social attachments plays a functional role in the development of sharing behaviors (Paulus, Becker, Scheub, and König 2016). Giving also reinforces prosocial behaviors by validating one’s identity as a capable, caring contributor to society (Grant and Dutton 2012). Markedly, the highest virtue in many Native cultures is generosity, a value that accords prestige and is embedded into many ceremonial practices (Brendtro et al. 1990/2002). The benefits of giving are well established in the literature, and research has shown that generative concern and generative action are associated with higher levels of life satisfaction, happiness, and psychological health (Wang, Tran, Nyutu, and Fleming 2014). In addition, culturally responsive programming that attends to the needs and interests of tribal communities have been found to be an important component of attaching meaning to Native students’ research experiences and providing a sense of self-worth (Ward et al. 2014).

Generosity is reflected in the larger purpose of the research that students are involved in through the SURE program. Although students have a diverse range of research projects to choose from, these studies are centered on the need to solve health problems, reduce health disparities, improve patient care, and promote health and well-being. Generosity is also advocated through opportunities for students to volunteer at science festivals, conferences, and other similar events along with options to present their research and share their knowledge and experiences with Native youth.

## Present study

Despite the problematic aspects of education described earlier, education can be a mechanism for cultural preservation, social mobility, and economic prosperity. However, Native Americans are drastically underrepresented in higher education (Executive Office of the President 2014). In fact, Native Americans have the lowest completion rates of a bachelor’s degree or higher in the U.S. compared to any other racial or ethnic group (Ross et al. 2012). Though educational access and retention are important components to reducing educational disparities, meaningful participation and having a voice in the narrative of academic knowledge production is equally important, if not more. Despite efforts by federal agencies and private organizations to increase the number of Native American scientists engaged in biomedical and social-behavioral research in the U.S., the recruitment of Native students in this field continues to be a challenge, and they remain severely underrepresented in these professions (Villarejo, Barlow, Kogan, Veazey, and Sweeney 2008).

Early exposure to scientific research has been cited as being effectual in attracting and retaining Native American students in the sciences and can act as a trajectory to graduate school and research activity, with mentoring being especially critical throughout this development (Gray and Carter 2012). However, mentoring is often in short supply, and this absence may be due, in part, to the challenges professors often face, such as a lack of support for mentoring from leadership at both the departmental and institutional levels as well as the extensive time commitment required (Prunuske, Wilson, Walls, and Clarke 2013). For those Native Americans who are engaged in academic research, they may find few (if any) other Natives among their immediate colleagues and frequently shoulder the responsibility of being “cultural translators”—negotiating, explaining, and justifying the value of Indigenous science to non-Native colleagues and audiences and/or Western science to tribal communities (Walters and Simoni 2009). Other challenges and barriers reported by Native research investigators include the marginalization of their research interests and discrimination and microaggressions within university and research systems (Walters and Simoni 2009).

The underrepresentation of Native Americans among scientific researchers is problematic for a number of reasons. For instance, a shortcoming of homogeneity in the scientific work force is the stifling of scientific knowledge progression. In fact, studies have evidenced that diversity in the research workforce broadens scientific inquiry, which can lead to better methods for addressing health issues affecting racial and ethnic minorities (National Academy of Sciences 2005). Another is related to unethical scientific practices that have taken place in the past and continue to take place today. Not only has science been used as a mechanism for justifying political and social agendas that have had damaging effects on Native peoples historically, but these logics continue in the present. For example, Tallbear (2013) notes the dangers of using DNA testing to establish or authenticate Indigenous identity and citizenship, which can potentially undermine concomitant political claims. However, by walking and talking together, Native and non-Native researchers and Indigenous and non-Indigenous knowledge systems might more successfully discover cures, treat illness, and promote wellness.

As previously stated, the overarching goal of the SURE program is to develop a cadre of Native Americans scientists and health science professionals engaged in biomedical research linked to reducing health disparities. While research has shown that undergraduate research experiences can be a fruitful pathway to the health professions (Lopatto 2010), evidence also supports training Native health workers as a key strategy to improving Native health outcomes as well as applying Indigenous pedagogy to promote experiential learning and professional development (Main, Nichol, and Fennell 2000). Taken together, an undergraduate research internship modeled on Native wisdom and providing mentorship and training to support Native students’ interests in health science research may be an effectual pathway to careers in research and health professions and eventual reduction in health disparities. Since few studies have published on the effectiveness of applying Indigenous pedagogies to undergraduate research programs, the present study adds to the literature by exploring the utility of the Circle of Courage as a model for cognitive apprenticeships aimed at fostering a positive scientific learning environment and enhancing the academic and professional development of Native students.

Mentors were drawn from a research institute under an integrated, non-profit health system (lead host institution), a public university, and a local Veteran’s Affairs health system, all of which are located in a rural, Northern Plains region. Students were paired with a mentor or mentoring team with mutual research interests and work collaboratively on a scientific research project throughout the summer. The vast majority of students’ time was spent performing hands-on research tasks, which varies depending on the focus of the research project, and if it is in the physical, biological, or social sciences. For example, students could spend most of their day counting cells, running polymerase chain reaction tests, slicing mouse brains, conducting literature reviews, collecting and entering survey data, coding qualitative data, or running statistical analyses. Students also participate in other activities unique to their lab, including lab meetings, scientific talks, and trainings. For the remainder of their time, students attended group events intended to provide them with opportunities to network with scientists and other students.

The SURE program also incorporated components which aligned with many of the recommendations for successful Indigenous environmental education programming described by Simpson (2002). For example, though none of the mentors self-identified as Native American, mentors were recruited who had knowledge and experience working with Native American peoples. Mentors also worked with students to help them identify the real-world applications of their research and relevance to their coursework. Research projects were process-orientated and incorporated applied and issue-based scientific approaches. Students also attended a seminar series led by a Native elder who served as the Director of the Native American Student Services department at a local university. At this seminar, students discuss traditional Lakota values (e.g., bravery, fortitude, sacrifice, generosity, and hope), which are shared by many Native peoples, and how each plays a role in our educational journey. His talk also highlighted how these values can be applied to research, education, and professions in a way that both recognizes the strengths of traditional Native cultures while also improving the health and well-being of Native peoples.

## Methods

### Participants

Students were recruited for SURE through a variety of methods. Information about the program was made available through flyers and publications as well as online and on social media. Recruitment materials were also sent to a list of over 150 contacts in the Northern Plains region with interests in Native American education and health. In addition, students were recruited in person at the American Indian Science and Education Society (AISES) annual meetings and at events held at regional universities and tribal colleges. As a result, 46 students applied to the program in 2012–2014. Of those, 22 students were awarded internship positions. Ten students participated in the program in 2012, eight students in 2013 (one of which was in the program in 2012), and seven students in 2014 (two of which were in the program in 2012). The present study sample included 20 students (15 females, 5 males), with a mean age of 24.2 years (*SD* = 6.8, range 18.9–45.3). All of the students self-identified as Native American (50% Native American only, 50% Native American and White) with 13 different tribal affiliations and hometowns in 8 different states. As an incentive to participate in this study and as a celebration of their completion of the SURE program, food was provided for participants prior to data collection.

### Design-based research

Design-based research (DBR) was used in the continual development and improvement of the SURE program. DBR, as it was envisioned by Brown (1992) and Collins (1992), is a form of learning in context which combines education research with theory-driven design to develop, enact, analyze, and redesign learning environments and theories of learning. DBR is a context-driven approach (or rather series of approaches) to engineering innovative learning theories and practices through evidence-based practices (Barab and Squire 2004). These learning designs help to generate new or revised theories about the processes of teaching and learning and improve educational practice. Consistent with a design-based research approach, the program underwent shifts in design based on findings from the previous year (The Design-Based Research Collective 2003). The ways in which coding supported the emergence of theorizing and informed how the program was periodically redesigned is described in the data analysis techniques section. The specific changes implemented will be outlined in the discussion.

### Data collection procedures and measures

The data collection and analytic methods used in this study reflect a qualitative methods design. Qualitative methods were selected for this particular study as this approach “gives voice” to the lived experiences, perspectives, and opinions of participants and provides depth to these contextualized accounts (Wendt and Gone 2012). This methodology also allowed us to—and to some extent, the reader—immerse ourselves in the cultural context of participants and reflect upon the meanings of participants’ accounts of their experiences. However, we also recognize the potential limitations of us, as non-Indigenous researchers, presenting data within the analyses as authentic Indigenous voices and acknowledge that data is interpreted through our own epistemological lenses and positionality.

The specific method of qualitative inquiry used was focus groups, which are collective interviews with a relatively homogenous group of individuals assembled by the researcher(s) to discuss personal experiences and/or opinions about a specific topic, product, or intervention (Powell, Single, and Lloyd 1996). Unlike interviews, focus groups rely on interactions between members of the group as part of the data collection process (Sagoe 2012). This methodology was used to elicit conversations and discussions with and between participants about their individual and collective experiences in the program.

The development of focus group questions was informed by the four quadrants of the Circle of Courage: belonging, mastery, independence, and generosity (see Table 1). Additional questions were also included to supplement pre/post survey data by providing a more in-depth understanding of interns’ program expectations, positive and negative experiences, program impact, and suggestions for improvement. At the close of focus group discussions, the co-moderator provided a summary of participants’ responses, with a follow-up question allowing participants the opportunity to provide additional input on topics they wanted to discuss that weren’t covered in the focus group. The data reported in this study is part of a broader study evaluating the impact of the SURE program. Though pre and post survey data were collected and analyzed (see Griese, McMahon, and Kenyon 2016), the present study focuses on the qualitative data.

Focus groups were held annually on the last day of the summer internship and were conducted in a private room at a research facility, with research staff (none of which mentored the students) moderating and co-moderating the discussions. Focus group discussions were audio recorded and transcribed verbatim. Audio length ranged from 83.8–100.6 min (*M* = 94.3 min). Participants were not compensated financially for their participation. However, as previously mentioned, food was provided. Informed consent was obtained for all study participants, and all study procedures were approved by both the non-profit health system and the university Institutional Review Boards.

### Data analysis techniques

Focus group transcripts were stored and analyzed using the Qualitative Solutions and Research International NVivo 10 software program (NVivo 2013). As recommended by Fonteyn, Vettese, Lancaster, and Bauer-Wu (2008), NVivo was also used to create an electronic database of the transcripts that had been coded using the consensus codebook and to update and revise the evolving codebook. Data were analyzed via deductive content analysis (Elo and Kyngäs 2008) in which a categorization matrix was developed with coding categories derived from the Circle of Courage themes. Coding categories were operationalized using code definitions and coding decision rules. The coder read the focus group transcripts, making notes on initial impressions, and coded the data in a manner consistent with code definitions and the parameters set for coding text. Data within these categories were then grouped within subcategories of comparable content. Data that did not fit within the categorization framework were open coded, and categories were generated which best described the data. Open codes were then reviewed, descriptors were added, and codes were further classified into conceptual categories of similar meanings and defined. This method of building new concepts supported the identification of emergent themes for later analysis.

Recurring team meetings were held for in-depth discussion of how the participants’ quotes fit (or did not fit) the meaning of the Circle of Courage themes. In the final application of codes, if debate remained among the team, the final decision was made by the principal investigator. All coded text was then reviewed and, if necessary, recoded to maintain consistency with the code definitions and coding decision rules. This method of analysis was chosen since the deductive process allows for an assessment of the theory and examination of how the theoretical constructs inherent to this model (belonging, mastery, independence, and generosity) impacted students. Results were then reviewed on an annual basis by the evaluator and example quotes representative of themes were pulled to summarize results. Summaries were reviewed by program staff and guided discussions regarding effective methods for applying the Circle of Courage in this context, the development of new theoretical understandings, and the iterative refinement and continuous evolution of the program design.

#### Trustworthiness of themes

To ensure the trustworthiness of themes, the recommendations of Lincoln and Guba (1985) and Graneheim and Lundman (2004) were followed for establishing credibility (truth value), transferability (applicability), and dependability (consistency). First, credibility was affirmed through the inclusion of both female and male participants of various ages, class standings, geographic backgrounds, tribal affiliations, and perspectives, thereby contributing to a more diverse range of programmatic interpretations. Credibility was further enhanced by cross-checking the preliminary themes among co-researchers (i.e. the principal investigator, program evaluator, and an investigator external to the SURE program). Although findings cannot be generalized to all students or settings, detailed descriptions of the program context, participants, analytic process, and representative examples of research findings are provided to strengthen transferability. Since data was collected over the course of 3 years, consistency in data collection (dependability) was assured through the uniformity in the focus group guide used for each cohort, with minor edits to one question and prompts to provide more clarity.

## Results

As previously mentioned, data were coded and categorized under the respective Circle of Courage constructs of belonging, mastery, independence, and generosity (see examples in Table 2). Analyses revealed a variety of ways in which the aspects of belonging, mastery, independence, and generosity built into the program had an impact on students.

### Belonging

Feelings of inclusiveness were evident in focus group responses, which were largely attributed to in-person interactions with their peers, feeling respected, and the accessibility, guidance, and investment of their mentors and laboratory team. Recommendations for expanding opportunities to belong were also provided and will be explored further in the discussion.

#### Interactions with peers

Having program activities that brought students together on a regular basis was pivotal to feeling as though they were a valued member of the larger group. As one student affirmed, “… Seeing each other all the time, that’s what really brought the camaraderie, to get more.” Students also mentioned that being part of a program such as this, specifically for Native students, was a significant aspect of the sense of community they experienced.

That’s what I would say, like, the biggest pro was-, was like, to me, growing into a program that was for all Native students. Like, having the community inside, that was really important for me, for the experience. So, like, getting to see everyone at least once a week—well, technically twice a week with the journal clubs—that was really important to me.

#### Cultures of respect

Moreover, being treated with respect and a contributing member of a research team nurtured feelings of significance.

I felt extremely respected as a person. And I felt like I was treated as an equal. Not that I was just an intern, but that my opinions and things mattered, and my input was wanted, um, and not just discarded once it was given. So that was, I mean, for me, that was a lot. That meant a lot.

Mentors and research team members having some familiarity with Native cultures was also instrumental to creating a welcoming environment, as highlighted by one student:

… My mentor and a lot of people in the lab that I worked at were fairly Native-orientated, and that made me feel a lot more welcome, that they weren’t completely oblivious to Native American stuff….

Working intimately with lab colleagues also led to some degree of introspection and self-discoveries among students.

I think that I learned that I like to work with people. So I think that was huge, because there’s a lot of, um, paths you can go into for med school where you don’t work with people….

#### Mentorship

Another important piece to feeling a sense of belonging was interns’ relationships with their mentors. While receiving guidance as they developed their scientific research skills was important, interns expressed appreciation for the fact that their mentors also cared about their well-being.

I think, like, my mentor, again, helped bring me in. And, um, one of the first weeks I was in the lab here, we went out to lunch to celebrate a couple different things…. Sometimes they would call me and just see how things were going. Like, not even with research, just, “How are you doing? Is everything okay?” And, um, just kind of checking in to make sure research wasn’t affecting other things in my life. That was kinda [*sic*] nice, showing that they cared.

Several interns also identified a team of mentors they were able to rely on throughout the program. This included other faculty members and lab members who, together, welcomed them to the program and their labs, working to ensure inclusivity. For example, one intern stated:

And I mean, all the mentors that I had made me really feel like I was, you know, a part of their full-time lab. Where they were like, “If you need anything.” And everyone in the labs were [*sic*] really helpful anytime I had a question. So, um, overall, it was just, like, for my experience, I was just really happy with how it turned out.

Another echoed this sentiment, saying:

The mentor I had was extremely welcoming, and I had guidance every day with a graduate student who was a PhD candidate. And, um, and it really aligned well with what I thought I needed for my own personal growth in science.

### Mastery

Cognitive advancements were also evident in focus group responses along with a growing confidence in their abilities to conduct scientific research. Interns suggested the experience compelled them to develop critical thinking skills and overcome obstacles and challenges. As one student revealed, “… It challenges you to push yourself, to kind of reach the next level and break through plateaus. And [in] that way, it was really cool.” Others mentioned the confidence they built throughout the summer as they worked with their mentors and alongside other students and lab members. As a result of their experience, they felt they were able to develop deeper scientific understandings, which led to a greater appreciation for the potential impact of their work, as evidenced by one student’s remarks:

… The confidence level definitely increases by just the fact that, you know, you feel more confident in yourself and what you are talking about. And in just that feeling, you know what you are saying is important to some people, that it can actually make a difference.

#### Experiential learning

Interns also highlighted the practical gains of being immersed in an experiential learning environment, an environment that differed from the lecture format they had grown accustomed to in the classroom. One intern expressed, “We were put into real labs, working with real people. We know how, just how they operate on a day-to-day basis, long after we leave the internship. And that real-world experience is really invaluable.” By contrast to the traditional classroom setting, engaging in research provided them with hands-on experiences which reinforced what interns had learned in the classroom.

… There are techniques in the biological lab that I was in that I had never done before. And, you know, you learn about them in class, but if you don’t have a lab, you don’t actually execute them. I mean, if you do, you don’t actually execute them to the level that would make you proficient at it, but you do here. And there is a level of intensity, because you are brought up to the professional level of the lab that you are in to become really proficient in these techniques. And, uh, and now you can add that to your resume or your CV, and that’s something really valuable.

#### Research process skills

Interns also frequently discussed the research process skills they were able to master throughout the research experience, suggesting that, while this experience allowed them to gain a broad spectrum of skills, it also gave them insight into the various stages and integral parts of a research study as well as the creative thinking science often requires.

… When I thought of a scientist, I thought of logic, by the book, and all this stuff. And it’s more. It’s interesting to see, as a part of my experience, the creative side of it. And like, coming up with how you’re going to test, and what you’re going to test, what are you gonna [*sic*] do about it, like, when you’re starting.

Such realizations led some students to have a greater appreciation and higher regard for science, as seen in this statement:

I feel like science is a lot bigger than I realized, and there’s, um, certain stages of research even. And I have a new found respect for science and how much time and effort is put into it. Because I’ve always, I read articles … and I think now when I read one, I’m like, wow, they had to have a lot of work put in to get that far. And I think it’s kind of finding out what you’re in for [inaudible], um, around for the bigger role of science.

They also discussed the discovery that unforeseeable events often take place throughout the research process, necessitating patience, adaptability, and ingenuity. As one student posited, “I guess, from what I’ve experienced, science is very unpredictable. So I could see how that would factor into needing to be flexible, um, and just rolling with the punches.” In addition, this experience helped students discern the utility of science.

I think one of the pros from me was actually realizing that all the hours that I sat in class were for something, … how much more what I learned in those classes fit in and made more sense.

More, they recognized the continuous need for science. One student discovered, “Even though there is already science out there, there is always room for improvement. And there’s, uh, a need, um, continuously, because there is [*sic*] new things that pop up all the time.”

#### Mentoring

Similar to feeling a sense of belonging, mentoring played a crucial role in advancing students’ cognitive proficiencies.

I feel like my mentor went above and beyond what he was supposed to do…. Like, every single thing that I wanted to know, he would go over it with me and make sure I understood it. And so now, when I had to present my poster, I felt so comfortable, because I knew everything that I needed to know to present that research….

In this case, the mentor was available and supportive, providing the student with sufficient knowledge, while also allowing them to learn at their own pace. As occasions arose wherein students had to explain their research to others outside their discipline or who were less knowledgeable of the project, being able to simplify the science led to an increase in their own level of understanding.

I kind of talked them through what I was doing and why I was doing it, and everything. Just kinda [*sic*] explain[ed] my project. And I had to do it on a level that was more, um, I guess, easier language, uh, where it wasn’t the scientific language. And it kinda [*sic*] brought me back down to being able to explain it to someone so it’s easy to understand. So that was really helpful, too, ‘cause [*sic*] it taught me to understand it better….

#### Career development

By having access to mentors and experts within the science field, many interns began to contemplate their academic and vocational path and seek meaningful advice.

I think it has helped to clarify my path further, so-, so not necessarily change, but more of a refinement of goals, um, because there’s access to so many professionals with so many different areas with expertise. And so you can grab any one of them after a seminar that we’ve had and ask them questions about, “Well, this is what I am thinking. What do you think?” And there is some really good advice that came out of that.

Participation in research also demonstrated its value. As one student commented, “I think, just going through the program, I would be more proud if I got a research job…” Another observed, “I’m actually, like, since I’m in education, I didn’t think about research whatsoever, but I feel like research is, like, in your everyday life. You need it….” For other students, research helped them recognize the versatility of a science degree.

… I was very content on changing my major away from science completely after this summer. [SURE] made me really focus and realize, what I’m going for, there are so many options, especially with my major.

Additionally, for some, this refinement of goals was a motivating force.

Like for me, I’m starting to get focused a little bit more with school and what I really wanna [*sic*] do. Going through this program helped me to keep my focus to go into research and gave me a lot of motivation.

‘It really helped to, helped me to find, or I guess, bring my majors together. ‘Cause [*sic*] I have two majors that are on two completely different ends of academia. One is a bachelor of arts and ones a bachelor of science, and there’s a lot of times people are like, ‘How are you gonna [*sic*] combine those two?’ Um, but after this, it was really great, because I can, um, do research that is, um, medical research in Native communities. And I have that background in both of them, so that I can, you know, have the qualifications to be able to do that. It’s helped to take what I was already interested in and, uh, kinda [*sic*] break it down into or, uh, shape it into what I wanted it to be.’

The research experience not only helped to clarify students’ educational and professional goals, but also provided motivation to continue with their schooling in order to fulfill their professional goals.

### Independence

Acquiring the confidence to be independent happened gradually through the use of instructional scaffolding coupled with self-regulated learning. Students were also able to acquire autonomous learning strategies, which gave rise to individual agency and the self-efficacy to carry out research tasks.

#### Instructional scaffolding

Crucial to developing autonomous learning strategies was the instructional scaffolding. Mentors provided students with not only the guidance they needed while familiarizing themselves with the project, but also reassurance throughout the learning process. One intern explained:

In my lab, being independent was really difficult at first, even though I had some experience last summer…. So just having someone there to reassure you that [you’re] doing things right, and then easing it off and being able to do it on my own and regaining the knowledge that I had. And like, oh yeah. I understand. I get it now.

One of the results of developing autonomous learning strategies was greater confidence in their abilities. In describing their ability to calculate a logistical growth curve typically run using statistical packages, one intern stated:

… I was like, I don’t [want to use a statistics package]. Let me try and do it, and I figured it out. And I come to find out that, one of the other mentors told me that was, like, Calculus III kind of stuff, and I hadn’t even taken Calculus II yet. So that made me really confident in that and myself, educating myself.

#### Self-regulated learning

The ability to self-direct their learning was also an effective instructional tool that helped facilitate independence.

I felt like, you know, rather than just saying, this is what we want you to do and this is how we want you to do it—which wouldn’t have taught me anything, you know—I learned a lot being independent.

Many of the students also felt as though they were able to accomplish the goals they had set for themselves at the beginning of the summer and documented in their ILPs. One intern reflected, “I feel like every goal I set out to accomplish this summer, I achieved….”

#### Independence as a researcher

The degree to which some students were engaged in research as active agents in the investigative process led to greater self-regard as independent researchers.

Overall, like, in a ten-week program, I’ve made a lot of progress. Where it’s like, even compared to not just this last year, but compared to two years ago I- I didn’t see myself with this much knowledge or, like, having my own project as successful as it’s been.

‘One of the pros for me was that, um, even though it was the first year I had gotten research, and stuff like that, I had actually made two procedures in my lab. So even just starting out in research, I mean, you can have a fairly big impact in the research.’

This pride also led to greater confidence as a self-determined investigator, as shown in this student’s revelation: ‘Um, I think just being in the program, in itself, gave me confidence, to be accepted and be able to hold these positions….’

#### Limited independence

While the majority of students were afforded a reasonable amount independence, there were some who felt their independence was stifled in some ways, either by their mentor or by the inability to develop their own, independent research project.

I heard, like, two people say that their mentors really dictated what they had on their posters and stressed them out…. ‘Cause [*sic*], I mean, it is the mentor’s research, kinda [*sic*], but it is also the person’s, too….’

‘I thought it would be kind of like our own…. And ours was just, like, helping on someone else’s [research project]. So I mean, it was important, but we really didn’t get to do our own research.

### Generosity

Although generosity was mentioned less frequently compared to other themes, the potential of their research to improve the lives and inspire the hopes and ambitions of others was recognized by students. For example, students often expressed a desire to extend their efforts to their families, home communities, and to Native people.

… My little brother is, I mean, he’s going to school right now, and hopefully there will be like, “Ok, I can do that, too, then.” I think all of us could be accounted for that, like, for Native people, in general, saying that we’re doing this. You know, like give confidence not only for ourselves, but the people back home.

Many realized that their impact could also cut across boundaries, seeing their potential impact on a larger scale. Before this experience, many students indicated they were unaware of research efforts being made to reduce health disparities. The SURE program helped them to not only be mindful of this issue and the work being done to address it, but also of the role they play in promoting health equity.

… I didn’t even know that this part of, like, health research and outreach really existed. So that really, like, opened my eyes to be like, oh, there are people out here who are, like, trying to help the health disparities in American Indians. And that really, like, opened my eyes and was like, wow. That made me, like, want to help more and be a part of that, so I think SURE did a great job in that.

There was also mention of how the impact of their research might not be seen immediately, making it difficult to gauge how their work might benefit others. Within a 10-week program, the effects may be minimal, as indicated in the following statement:

I don’t, I mean, we do a lot, but we don’t make any big changes in two months. Like, I mean, we do a lot of work, but it’s, you know, something that takes more time than just two months to make a big change.

However, some students held optimistic views about the prospective ways in which their work might help others.

… I mean, think about some of the research that we’ve done, and in an inverted way, it really helps people. And so you’re really affecting other people’s lives. It might not be happening now, but one day it will reach them.

## Discussion

Findings indicate that mentors played a critical role in cultivating relational attachment and significance, competency, achievement, and self-determination within a communal learning environment, a finding that is consistent with the broader literature (e.g., Rieske and Benjamin 2015). The important role of mentors in building relationships of belonging was evident in that interns often noted the significance of their mentors having cultural awareness and respect, displaying sincere concern for their well-being and personal growth, creating a welcoming and inclusive environment, and establishing personal connections with their peers. Conversely, the absence of cultural competency and regard can have adverse effects, as pointed out by one student:

… ‘Cause [*sic*] my mentor, after the ILP [Individualized Learning Plan meeting], was like, she crossed out the Circle of Courage model and, like, wrote, like, different, like, goals that she wanted to reach. And, I don’t know, I felt like that was disrespectful. And I was like, “Okay. How do I even go about saying that that this is like a model that we go off of, and there’s more to this program than just me and you?” But I didn’t know how to, like, say anything, so I didn’t. And so, I think, if she, if they even knew what, more about what it was about, that probably wouldn’t happen.

Once we became aware of this issue, which appeared to be an isolated incident, more concerted efforts were made to advise mentors on the background and use of the Circle of Courage while also conveying its importance to nurturing a strengths-based learning environment students can flourish in. The happenings recounted by the student also exposed power differentials whereby the student felt constrained to confront their mentor about her or his disregard of the Circle of Courage. Correspondingly, one of the SURE interns was hired on as a full-time employee, working closely with students as a peer mentor and program coordinator and providing students with a trusted confidant and additional support.

Other changes made to the program since its inception relate to expanding opportunities to belong. Given the logistic challenges of fostering social cohesion between students working at various sites, some felt disconnected to the other students who weren’t working at their site. Most of the recommendations to overcome this barrier focused on incorporating a day every week where students from each site could get together.

What I was going to suggest, is that Thursday, um—I think that’s when I had a lot of cubicle time—and I think that to increase belonging, maybe make Thursday an all-day SURE thing. Like, all your hours are SURE that day. Because it’s, I mean, it’s essential [inaudible], because you can’t do too much in three hours in a lab. So maybe, maybe every Thursday, have a social thing out every Thursday.

Therefore, starting in the second year of the program, students got together at the lead host institution for a full day each week to attend a seminar series, lead journal club discussions, and tour labs. Focus group responses after the incorporation of these changes suggest that belonging increased, lending further support to the importance of face-to-face interactions as a means of building close relationships. For example, one student who was in the program two consecutive years commented:

… The positive of this summer from last summer, is that the seminar series and everything and the journal club was done together. So it made a lot of people [have] a lot more person-to-person, quality relationships between, um, the [university site] and the [health system site] students that are in the program, and it allowed for more connection between, like, the students.

Taken together, findings suggest that, through the application of the Circle of Courage, the SURE program was successful in fostering an inclusive, positive peer culture that facilitated growth in students’ cognitive knowledge and autonomous learning strategies, while simultaneously refining and strengthening education and vocational goals, motivation, and focus. As such, this study supports the use of this framework in research experiences for Native students and the efficacy of such experiences in advancing student learning. Therefore, it is recommended that science education courses and programs involving Native students utilize Indigenous pedagogical approaches, such as the Circle of Courage, along with cognitive apprenticeship learning strategies (e.g., self-regulated learning, instructional scaffolding, and experiential and problem-based learning).

This study also helped to discern potent methods for nurturing collective learning experiences of mutual reciprocity. As an integral member of a research team, SURE students had a voice in the production of scientific knowledge. By building these relationships between Native students and scientists, students received guidance and encouragement from their mentors and support from their affiliated institutions. Scientists and science itself can benefit from working with Native students through the expansion of ethical and ideological considerations, thereby contributing to more robust scientific methods and theories. While not a focus group theme, this reciprocal relationship was brought up by one of the participants:

I think that, going back to him [another focus group participant] mentioning [SURE program staff], they helped bring up that topic, too, of like—I was working with Native American tissue samples, so they were asking me how I felt about what happens to those afterwards, um, and everything. It kind of helped me to lead into that discussion with my mentors, because, um, with some Native communities, it’s really important what happens with those, because, you know, it’s part of someone. So it was really awesome, so that we can kind of bring that back and have that in mind and bring that back to some of our mentors. Like, it’s one of those things where they didn’t really completely understand that or see that side. So it was nice just to be able to have that conversation.

Hence, it is reasoned that the relationship between students, researchers, and scientific disciplines is reciprocal as opposed to a means of acculturating or assimilating Indigenous students into science research.

## Limitations

Since participants were not directly asked about politics and power dynamics, this study is limited in its ability to speak directly to the potential dynamics at play and the degree to which the program was situated within an interstitial space. Furthermore, focus groups did not include questions about students’ unique positions on their research projects, including the research process, data collection procedures, hypotheses, analytic interpretations, or ethical practices. While this topic was brought up by one of the students, it cannot be ascertained whether or not such epistemological, methodological, or ethical questioning occurred among other program participants.

Other limitations of this study can be attributed to the use of focus group methodology. Data was collected over the course of three summers of the SURE program with a small cohort of students participating in the program each summer. Consequently, there was a relatively small sample size, limiting our ability to explore comparisons between students (e.g., tribal affiliation, age, gender, academic level). Though varying tribal affiliations were represented, participants were not a representative sample of Native American tribes. Since there are currently more than 567 federally recognized tribes in the U.S. (U.S. Department of the Interior 2017a) and hundreds of non-federally recognized tribes, coupled with the fact that both individual tribes and individuals within tribes can differ greatly from one another, these research findings are not intended to be generalized to all Native American peoples. That being said, given this study’s focus, findings could serve as the basis for the development of undergraduate research experiences for Native students.

While focus group methodology can be an effective means of gathering data that cannot be easily studied using quantitative methods, scholars have also noted challenges associated with this methodology. Constraints which may confound the data generated in focus groups include the potential for the group to be influenced by one or two dominant people, holding focus groups in a partisan environment, and the influence of social and group norms on participants’ comfort in voicing dissent or sharing personal information (Sagoe 2012). Though procedures were followed to avoid these circumscriptions (e.g., selecting moderators with experience and training in conducting focus groups), some participants may have felt stifled by group dynamics or lacked confidence in their ability to articulate their point of view and thus withheld sharing their personal experiences or opinions.

As is the case with other social scientific methods, interpretive analyses of focus group data require a considerable amount of judgment and care. Responses to focus group questions, at times, elicited responses with several underlying themes. Consequently, some discretion was used to determine which theme best fit the intended meaning of the response within the context of the discussion. However, this contextual overlap is also a testament to the richness of qualitative data, and rigorous methods were used to make consistent, objective decisions in the interpretation of data. For example, themes were defined and decision rules were applied to safeguard against inconsistencies in the interpretation of data. This also provided a method for cross-checking data to ensure our understanding of participants’ statements accurately represented the information provided by participants, that the implicit meaning of those responses was not invented by the focus group moderator or coder.

## Conclusion and future directions

This research provides encouraging findings about the benefits of engaging Native students in undergraduate research and the effectiveness of applying an Indigenous model to such programs. Results are also promising given that, at the national level, Native Americans continue to be underrepresented in science and science-related occupations. To ensure that Native students have equal access to these opportunities, there is a need to expand undergraduate research programs with an Indigenous focus. However, for undergraduate research experiences to move beyond targeted recruitment strategies, careful consideration should be paid to the place, context, and cultures in which students learn. The findings of this study indicate that the Circle of Courage can provide a useful framework for such programs, including: (1) providing students with opportunities to interact with their peers, mentors, and research team in both formal and informal settings; (2) underscoring the importance of working with students as respected and valued members of a team; (3) exposing students to a diverse range of research and academic professionals; (5) utilizing experiential and self-regulated learning strategies coupled with instructional scaffolding; (6) and highlighting the potential impact of their research and ways in which the knowledge and skills gained can be used to serve their tribe and/or home community.

Additionally, as reasoned by Bang and Medin (2010), science education should support students’ navigation of multiple epistemologies which take into account Native ways of knowing and ways of being. In the identification of potential topics for investigation, it is important to align research with the interests and needs of the Native students who will be working on these projects as well as those of tribal communities. This can be a key component to what Ward et al. (2014) refers to as attaching meaning to their experience. One of the ways in which projects and undergraduate research experiences can be more inclusive of tribal communities is through the incorporation of tribal participatory research projects and expanding undergraduate research experiences at tribal colleges.

When data was collected for this study, SURE did not include comprehensive participation of tribal community members, a potential mechanism for program expansion to promote tribal self-determination. SURE is currently in its seventh year of programming and has been expanded in recent years to include two reservation sites, the outcome of a community-based participatory research (CBPR) partnership with a tribal college and the host institution having an office on a reservation. This expansion has led to the incorporation of tribally-led programs and research projects, opportunities to work with Native mentors, and the incorporation of Indigenous ways of knowing and ways of being.

CBPR—also referred to as Tribal Participatory Research (TPR)—has become an increasingly popular approach for involving Indigenous communities and tribes. This methodology advocates for the shared ownership and partnership of community members and tribes in all stages of the research process and may be effectual in building partnerships with tribes to support and sustain the growth and development of Native researchers and create opportunities for Native students to work with Native mentors on tribally-driven research projects. It should be noted, however, that CBPR/TPR may be limited in its ability to equalize power relations and, in some cases, can reproduce or reinforce existing power dynamics. For instance, power differentials can be perpetuated in CBPR/TPR practices through the hierarchical positioning of dominant worldviews above the community members researchers are working with, formal and informal bureaucratic structures and institutions can sanction researchers as more powerful than the community members involved, and the ideals of this methodology may not be upheld if proper monitoring and evaluation practices are not in place (Darroch and Giles 2014). However, by recognizing and exposing the ways in which power differentials are perpetuated, utilizing postcolonial theories, and partnering with tribes and community members in all stages of the research process, CBPR/TPR can have an active role in advancing research that espouses tribal self-determination (see Hallett et al. 2017 for more specific recommendations for developing and executing community-based qualitative data methodologies with Indigenous peoples). Therefore, we suggest CBPR/TPR as a potentially useful method for those involved in efforts to engage Native students in tribally-driven research projects.

Undergraduate research experiences provide essential learning opportunities and academic preparation for higher education. Specifically, these findings demonstrate how engagement in research can advance students’ interests in science and improve their confidence in their academic abilities. Given that few studies have published their findings investigating successful strategies for supporting the academic development and retention of Native students, there is a continued need to share knowledge related to the assessment of these strategies in a variety of forums. Furthermore, as mentioned earlier, historical and more recent cases of unethical research with Native populations have generated justifiable mistrust of research in many Native communities. Building collaborative relationships with mentors and involving Native students in research as investigators rather than subjects may be a salient mechanism for establishing and restoring trust, further highlighting the need to expand opportunities for Native students to engage in research.

## Figures and Tables

**Table 1:** Focus group questions used for data analysis

**Warm Up Question:**
Having gone through the SURE program, what do you think it would be like to be a scientist?
**Thinking of your experience in the SURE program**:
Thinking back to before you started the SURE program, what were some of your initial expectations about the program? Which expectations were met? Which ones were not met?
What were some positive aspects about the SURE program?
What were some aspects about the SURE program that could be changed or improved?
**Now we are switching gears to talk more about the specifics of the SURE program**:
Tell me about your satisfaction or dissatisfaction with the program staff (e.g. mentors, PI, weekly speakers, etc.)
For this next question, I would like all of you to refer to the handout listing each of the weekly seminar sessions…. Which were your favorites and why? Which were your least favorites and why?
As some of you know, we have made some changes to the SURE program in the past year. Some of these included [list of recent changes]. What do you think of these changes?
**Now we’d like to talk more about the abstract goals of the program**:
How well did the program promote a sense of belonging between you and the other SURE participants? Between you and your research team/lab?
Thinking back to when you first started the program, how well did the program increase your confidence in your abilities to do research?
How well did the program facilitate your confidence in your abilities to do research tasks without supervision/guidance?
In what ways did the program help you better clarify your educational and career paths, even if this meant deviating from the education/career path you had in mind prior to this experience?
In what ways were you given the opportunity to share your knowledge and skills with others? Opportunities to help teach/lead others?
Now that you have completed the program, how do you feel about your personal progression throughout the summer? Do you feel that you got to accomplish the things you wanted to?
In what ways did the program increase your knowledge of health disparities and health disparities research?
In what ways did the program increase your knowledge of how biomedical research fits into the broader health and education systems?
**Now we’d like to hear how you would recruit Native students into the SURE program**:
What are some suggestions you have for getting other Native students interested in SURE?
**Now we’d like to hear suggestions for expanding the program to include other sites**:
We are considering expanding the SURE program in future years to include other sites in the Northern Plains area. What suggestions do you have for facilitating a sense of belonging amongst participants without physically connecting with them?

**Table 2:** Themes and example responses

Themes	Example responses
Belonging	“It’s more like a, opportunity is one way to say it, but that doesn’t necessarily give it enough credit, because it’s like, you know, there’s a sense of community and all this other stuff that goes into it.”
Mastery	“I used uh, well, I had to start and figure out and learn the process for like an IRB approval, um, kinda [*sic*] go through that, so that was interesting. But then I had to learn how to use a program which was called SAS, which really was a very confusing program, ‘cause [*sic*] it’s all a bunch of code that you have to put in. And it was a challenge, but I got through it. And then also like learning some of the new staining techniques, just kind of seeing how long or that the time that it takes to do those procedures. Because there was one staining that I did last year, but this year I think I did a couple more, uh, so just being able to extend that.”
Independence	“I learned a lot because they were, they helped me to be independent. They kind of just threw me into things, and I liked that….”
Generosity	“… it’s very technical, but me, myself, I’m trying to make it not that technical, and that’s the way I’ll use it to help other people.”

## References

[R1] Ahenakew C (2016). Grafting Indigenous ways of knowing onto non-indigenous ways of being: The (under-estimated) challenges of a decolonial imagination. International Review of Qualitative Research.

[R2] Ambtman R, Hudson S, Hartry R, Mackay-Chiddenton D (2010). Promoting system-wide cultural competence for serving Aboriginal families and children in a midsized Canadian city. Journal of Ethnic & Cultural Diversity in Social Work.

[R3] Bang M, Medin D (2010). Cultural processes in science education: Supporting the navigation of multiple epistemologies. Science Education.

[R4] Barab S, Squire K (2004). Design-based research: Putting a stake in the ground. Journal of the Learning Sciences.

[R5] Bartlett CM, Marshall A, Marshall M (2008). Facilitating the “talking and walking together” of Indigenous and mainstream sciences.

[R6] Bartlett C, Marshall M, Marshall A (2012). Two-eyed seeing and other lessons learned within a co-learning journey of bringing together Indigenous and mainstream knowledges and ways of knowing. Journal of Environmental Studies and Sciences.

[R7] Baumeister RF, Leary MR (1995). The need to belong: Desire for interpersonal attachments as a fundamental human motivation. Psychological Bulletin.

[R8] Blackstock C (2011). The emergence of the breath of life theory. Journal of Social Work Values & Ethics.

[R9] Brendtro LK, Brokenleg M, Van Bockern S (1990/2002). Reclaiming youth at risk: Our hope for the future.

[R10] Brendtro LK, Brokenleg M, Van Bockern S (2013). The Circle of Courage: Developing resilience and capacity in youth. International Journal for Talent Development and Creativity.

[R11] Brendtro LK, Brokenleg M, Van Bockern S (2014). Environments where children thrive: The Circle of Courage model. Reclaiming Children & Youth.

[R12] Brendtro LK, Mitchell ML, Jackson WC (2014). The Circle of Courage: Critical indicators of successful life outcomes. Reclaiming Children & Youth.

[R13] Brokenleg M (2012). Transforming cultural trauma into resilience. Reclaiming Children & Youth.

[R14] Brokenleg M, Van Bockern S (2003). The science of raising courageous kids. Reclaiming Children & Youth.

[R15] Brown AL (1992). Design experiments: Theoretical and methodological challenges in creating complex interventions in classroom settings. Journal of the Learning Sciences.

[R16] Cajete G (1994). Look to the mountain: An ecology of indigenous education.

[R17] Cajete G (2000). Native science: Natural laws of interdependence.

[R18] Cheek DW, Quiriconi M (2011). The role of state education departments in science education policy development.

[R19] Churchill W (2004). Kill the Indian, save the man: The genocidal impact of American Indian residential schools.

[R20] Collins A, Scanlon E, O’Shea T (1992). Toward a design science of education. New directions in educational technology.

[R21] Cram F, Phillips H (2012). Claiming interstitial space for multicultural, transdisciplinary research through community-up values. International Journal of Critical Indigenous Studies.

[R22] Crawford DM, Cheadle JE, Whitbeck LB (2010). Tribal vs. public schools: Perceived discrimination and school adjustment among Indigenous children from early to mid-adolescence. Journal of American Indian Education.

[R23] Darroch F, Giles A (2014). Decolonizing health research: Community-based participatory research and postcolonial feminist theory. Canadian Journal of Action Research.

[R24] Deloria E (1998). Speaking of Indians.

[R25] DeSalvatore G, Millspaugh C, Long C (2009). A journey from coercion to building courage. Reclaiming Children & Youth.

[R26] Elo S, Kyngäs H (2008). The qualitative content analysis process. Journal of Advanced Nursing.

[R27] Erikson EH (1950). Childhood and society.

[R28] Espiner D, Guild D (2010). Growing a Circle of Courage culture: One school’s journey. Reclaiming Children & Youth.

[R29] Executive Office of the President (2014). 2014 Native youth report.

[R30] Flynn SV, Duncan K, Jorgensen MF (2012). An emergent phenomenon of American Indian postsecondary transition and retention. Journal of Counseling & Development.

[R31] Fonteyn ME, Vettese M, Lancaster DR, Bauer-Wu S (2008). Developing a codebook to guide content analysis of expressive writing transcripts. Applied Nursing Research.

[R32] Forbes JD (2000). The new assimilation movement: Standards, tests, and Anglo-American supremacy. Journal of American Indian Education.

[R33] Frankowski B, Duncan P (2013). Always searching for strengths: Interviewing and counseling with the Circle of Courage. Reclaiming Children & Youth.

[R34] Garfat T, Van Bockern S (2010). Families and the Circle of Courage. Reclaiming Children & Youth.

[R35] Gentry M, Fugate CM, Wu J, Castellano JA (2014). Gifted Native American students: Literature, lessons, and future directions. Gifted Child Quarterly.

[R36] Graneheim UH, Lundman B (2004). Qualitative content analysis in nursing research: Concepts, procedures and measures to achieve trustworthiness. Nurse Education Today.

[R37] Grant A, Dutton J (2012). Beneficiary or benefactor: Are people more prosocial when they reflect on receiving or giving?. Psychological Science (0956-7976).

[R38] Gray JS, Carter PM (2012). Growing our own: Building a Native research team. Journal of Psychoactive Drugs.

[R39] Griese ER, McMahon TR, Kenyon DB (2016). A research experience for American Indian undergraduates: Utilizing an actor-partner interdependence model to examine the student-mentor dyad. Journal of Diversity in Higher Education, Advance online publication.

[R40] Gundlach Graham A (2012). The power of boarding schools. American Educational History Journal.

[R41] Hallett J, Held S, McCormick AKHG, Simonds V, Real Bird S, Martin C, Simpson C, Schure M, Turnsplenty N, Trottier C (2017). What touched your heart? Collaborative story analysis emerging from an Apsáalooke cultural context. Qualitative Health Research.

[R42] Harper E (2005). A Circle of Courage level system in day treatment. Reclaiming Children & Youth.

[R43] Higgins M (2014). De/colonizing pedagogy and pedagogue: Science education through participatory and reflexive videography. Canadian Journal of Science, Mathematics and Technology Education.

[R44] James AB, Renville T (2012). Ohiyesa’s path: Reclaiming Native education. Reclaiming Children & Youth.

[R45] Kierans K (2003). Mi’kmaq craftsman preserves ‘old ways’. The Halifax Sunday Herald.

[R46] Kim Y (2010). The procrustes’ bed and standardization in education. Journal of Thought.

[R47] Larson S, Brendtro L (2000). Reclaiming our prodigal sons and daughters: A practical approach for connecting with youth in conflict.

[R48] Lerner LS, Goodenough U, Lynch J, Schwartz M, Schwartz R (2012). The state of state science standards: 2012.

[R49] Lincoln YS, Guba EG (1985). Naturalistic inquiry.

[R50] Lopatto D (2010). Undergraduate research as a high-impact student experience. Peer Review.

[R51] Main D, Nichol R, Fennell R (2000). Reconciling pedagogy and health sciences to promote Indigenous health. Australian and New Zealand Journal of Public Health.

[R52] Marchand DM (2011). Circle of Courage infusion into the Alberta Indigenous Games 2011. Reclaiming Children & Youth.

[R53] Margolis E (2004). Looking at discipline, looking at labour: Photographic representations of Indian boarding schools. Visual Studies.

[R54] Maslow AH (1971). The farther reaches of human nature.

[R55] McKinley B, Brayboy J (2005). Toward a tribal critical race theory in education. The Urban Review.

[R56] Meza N (2015). Indian education: Maintaining tribal sovereignty through Native American culture and language preservation. Brigham Young University Education and Law Journal.

[R57] Morse WC (2008). Connecting with kids in conflict: A life space legacy.

[R58] Nadasdy P (1999). The politics fo TEK: Power and the ‘integration’ of knowledge. Arctic Anthropology.

[R59] National Academy of Sciences (2005). Assessment of NIH minority research and training programs: Phase 3.

[R60] NVivo An overview of NVivo.

[R61] Oakes AT, Craven R, Bodkin-Andrews G, Mooney J, Craven R, Bodkin-Andrews G, Mooney J (2013). ‘Not an educational institution’: Native American boarding schools in the 19th and 20th centuries. Indigenous peoples.

[R62] O’Sullivan L, Berthier-Foglar S, Collingwood-Whittick S, Tolazzi S (2012). Material legacies: Indigenous remains and contested values in UK museums. Biomapping Indigenous peoples: Towards an understanding of the issues.

[R63] Patterson DA, Wolf S, Ahuna KH, Tinnesz CG, Vanzile-Tamsen C (2014). Using self-regulated learning methods to increase Native American college retention. Journal of College Student Retention: Research, Theory and Practice.

[R64] Paulus M, Becker E, Scheub A, König L (2016). Preschool children’s attachment security is associated with their sharing with others. Attachment & Human Development.

[R65] Peacock TD, Cleary LM (1998). Collected wisdom: American Indian education.

[R66] Peat D (2002). Blackfoot physics: A new journey into the Native American universe.

[R67] Piaget J, Apostel L, Berger G, Briggs A, Michaud G (1972). The epistemology of interdisciplinary relationships. Interdisciplinarity: Problems of teaching and research in universities.

[R68] Powell RA, Single HM, Lloyd KR (1996). Focus groups in mental health research: Enhancing the validity of user and provider questionnaires. International Journal of Social Psychology.

[R69] Prunuske AJ, Wilson J, Walls M, Clarke B (2013). Experiences of mentors training underrepresented undergraduates in the research laboratory. CBE - Life Sciences Education.

[R70] Rauland C, Adams T (2015). A stronger smarter future: Multicultural education in Australia. Reclaiming Children & Youth.

[R71] Richards RJ (1993). Ideology and the history of science. Biology and Philosophy.

[R72] Rieske LJ, Benjamin M (2015). Utilizing peer mentor roles in learning communities. New Directions for Student Services.

[R73] Roberts JL (2000). Wilderness, a circle of courage, and the wisdom of elders—guiding the development of young people at risk.

[R74] Rosebud Sioux Tribe (2016). Welcome to the Rosebud Sioux Tribe.

[R75] Ross T, Kena G, Rathbun A, KewalRamani A, Zhang J, Kristapovich P (2012). Higher education: Gaps in access and persistence study (NCES 2012-046).

[R76] Sagoe D (2012). Precincts and prospects in the use of focus groups in social and behavioral science research. Qualitative Report.

[R77] Simpson L (2002). Indigenous environmental education for cultural survival. Canadian Journal of Environmental Education.

[R78] Spring J (2011). The politics of American education.

[R79] Tallbear K (2013). Native American DNA: Tribal belonging and the false promise of genetic science.

[R80] The Design-Based Research Collective (2003). Design-based research: An emerging paradigm for educational inquiry. Educational Researcher.

[R81] U.S. Department of Education (2017). The federal role in education.

[R82] U.S. Department of the Interior. B. o. I. Affairs (2017a). Bureau of Indian Affairs (BIA).

[R83] U.S. Department of the Interior. I. Affairs (2017b). The Bureau of Indian Education (BIE).

[R84] Van Bockern S, McDonald T (2012). Creating Circle of Courage schools. Reclaiming Children & Youth.

[R85] Villarejo M, Barlow AEL, Kogan D, Veazey BD, Sweeney JK (2008). Encouraging minority undergraduates to choose science careers: Career paths survey results. CBE Life Sciences Education.

[R86] Villarreal JM (2011). A magnet middle school longitudinal case study of student achievement, attitudes, and parental engagement. Doctoral dissertation.

[R87] Walters KL, Simoni JM (2009). Decolonizing strategies for mentoring American Indian and Alaska Natives in HIV and mental health research. American Journal of Public Health.

[R88] Wang MC, Tran KK, Nyutu PN, Fleming E (2014). Doing the right thing: A mixed-methods study focused on generosity and positive well-being. Journal of Creativity in Mental Health.

[R89] Ward C, Jones KW, Coles R, Rich L, Knapp S, Madsen R (2014). Mentored research in a tribal college setting: The Northern Cheyenne case. Journal of Research in Rural Education.

[R90] Wendt DC, Gone JP, Nagata DK, Kohn-Wood L, Suzuki LA (2012). Decolonizing psychologic inquiry in American Indian communities: The promise of qualitative methods. Qualitative strategies for ethnocultural research.

